# Carbon Nanocones with Curvature Effects Close to the Vertex

**DOI:** 10.3390/nano8080624

**Published:** 2018-08-17

**Authors:** Barry J. Cox, James M. Hill

**Affiliations:** 1 School of Mathematical Sciences, University of Adelaide, Adelaide , SA 5005, Australia; barry.cox@adelaide.edu.au; 2 School of Information Technology and Mathematical Sciences, University of South Australia, Adelaide, SA 5000, Australia

**Keywords:** carbon nanocones, geometry, curvature effects, mathematical modelling, approximate formulae

## Abstract

The conventional rolled-up model for carbon nanocones assumes that the cone is constructed from a rolled-up graphene sheet joined seamlessly, which predicts five distinct vertex angles. This model completely ignores any effects due to the changing curvature, and all bond lengths and bond angles are assumed to be those for the planar graphene sheet. Clearly, curvature effects will become more important closest to the cone vertex, and especially so for the cones with the smaller apex angles. Here, we construct carbon nanocones which, in the assembled cone, are assumed to comprise bond lengths and bond angles that are, as far as possible, equal throughout the structure at the same distance from the conical apex. The predicted bond angles and bond lengths are shown to agree well with those obtained by relaxing the conventional rolled-up model using Lammps software (version: 11 September 2008). The major objective here is not simply to model physically realisable carbon nanocones for which numerical procedures are far superior, but rather, to produce an improved model that takes curvature effects close to the vertex into account, and from which we may determine an analytical formula which represents an improvement on the conventional rolled-up model.

## 1. Introduction

Conventional models for carbon nanocones propose a construction comprising a sheet of graphene with a section removed which is then rolled and joined seamlessly to form a conical nanostructure [1]. Hereafter, we refer to this as the “rolled-up model”. Closed nanocones may have one of five conical angles which are determined by the amount of the graphene sheet that is removed and this, in turn, determines the number of pentagonal rings required to close the vertex of the cone [2]. The conventional models for both carbon nanotubes and nanocones assume that they comprise rolled-up graphene sheets that are joined seamlessly to form complete structures, and any effects arising from the changing curvature and bond bending and distortion are completely ignored. For carbon nanotubes, the present authors proposed a polyhedral model [3,4] which properly incorporates a hexagonal framework in which the bond angles and bond lengths are all assumed to be identical in the cylindrical configuration, and by necessity, the sum of the bond angles is less than 360°. In this paper, we propose a corresponding model for carbon nanocones, but in this case, it is not possible to produce a completely analogous model, since the present structure does not have precise equality of all bond lengths and bond angles since the curvature changes along the length of the nanocone and so too, does the angle sum of the three bond angles at each carbon atom. Therefore, evidently, it is not expected that every point in the graphene lattice will be exactly congruent with all others.

In previous studies on the structure of nanocones, there has been considerable interest in the geometry and morphology of the vertex for various cone angles [5,6,7,8,9], their electronic properties [10,11,12], and their mechanical behaviour [13,14,15,16,17]. However, in the modelling presented here, we concentrate on accounting for the relevant curvature effects along the wall of the nanocone, both close to and further away from the conical vertex. We derive an analytical expression for the cone radius that is applicable at any distance along the cone wall, and we also derive an integral expression for the conical height which goes some way towards accounting for the varying curvature of the cone wall. An asymptotic expansion of the integral expression gives the conventional rolled-up formula as the leading term, we may view the higher order terms in the series as higher order corrections to the rolled-up model. The predictions of this model are compared to molecular simulation results performed by the authors using the Lammps software package (see: https://lammps.sandia.gov) [18].

We comment that the assumptions adopted here for a symmetrical structure involving equal bond lengths and bond angles evidently ignore two important issues. Firstly, such a symmetric structure may not be physically realisable. Secondly, there may be other physical effects close to the vertex that violate the symmetry assumption and the bond length and bond angle equality. However, the major objective of the modelling presented here is to determine a mathematically tractable model that gives rise to analytical formulae which represent an improvement to the conventional rolled-up model. We do not claim that this is a universal model that can be applied to all physically realisable carbon nanocones, but rather, merely a step forward towards the determination of an analytical solution of what is, after all, a fundamentally difficult problem. Possible future directions in this regard include developing more quantitatively accurate methods to optimise the nanocone structure. For example, packages such as Qmcpack [19] or Casino [20] could be considered for this purpose.

## 2. Methods

To assess the usefulness of the model proposed here, we need to carefully measure the cone geometry. As there is, at present, no known technique to do such measurements through experiments, the approach adopted here is to formulate a geometric model of these structures and then compare the predictions with those obtained from numerical molecular modelling (Lammps). This approach is described in detail in Section 3 and Section 4. The method employed involves the determination of a set of atomic positions based on a bond length of σ=1.3978 Å, and the rolled-up model described in Section 3 is used to determine initial atomic locations. The simulated cones comprise n=2 panels, as described in Section 3. The panels are extended to 40 rows of carbon atoms and the atoms located closer than r=1.667 Å from the conical axis are excluded from the simulation which means that the conical cap is excluded from these simulations. Finally, the dangling bonds on both ends of the cone (base and vertex) bond with hydrogen atoms to stabilise the overall integrity of the structure for the duration of the simulation.

Using the cone established using the rolled-up geometry, the atom positions are loaded into Lammps, and the simulation is run to relax the structure, producing a more energetically favourable structure. This is done by starting the simulation at a temperature of 600 K and reducing it as close as possible to 0 K over 10,000 time steps using the adaptive intermolecular reactive empirical bond order (AI-REBO) potential. The AI-REBO potential comprises three terms and may be represented by the formula

$$\begin{array}{c} E=\frac{1}{2}\sum_{i}\sum_{j\neq i}\left[E_{ij}^{\mathrm{REBO}}+E_{ij}^{\mathrm{LJ}}+\sum_{k\neq i,j}\sum_{\ell \neq i,j,k}E_{kij\ell }^{\mathrm{TORSION}}\right]. \end{array}$$

For more details, we refer the reader to Brenner et al. [21]. After the simulation is complete, the final atomic locations are extracted and the conical radius for an atom is determined by measuring the distance between an atom on one panel and the matching atom on the second panel. The location of individual carbon atoms in the resulting structure is then analysed and compared with both the initial rolled-up structure as well as the structural predictions of the new model, as described in Section 4.

## 3. Theory

### Rolled-Up Model Formulation

**Figure 1:**
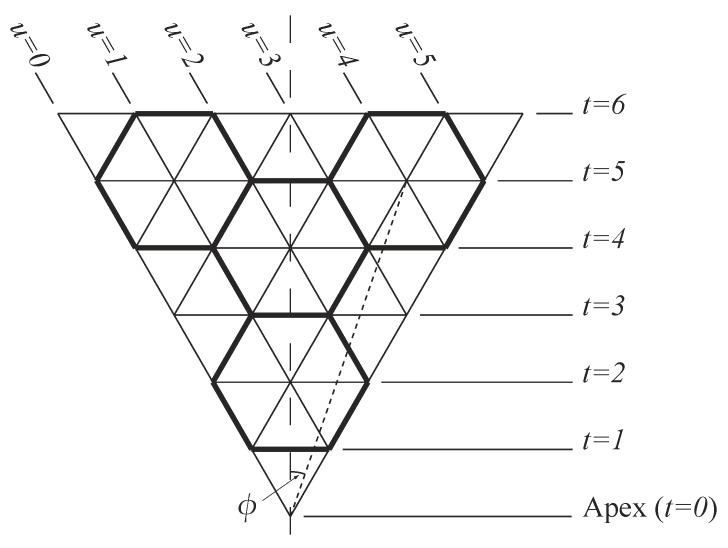
Geometry of a single panel (nanocones comprise from one to five such panels mapped onto a right circular cone).

In this section, we describe the conventional rolled-up model for carbon nanocones which may be used to provide a first approximation to determine the atomic positions, bond lengths, and bond angles in such structures. In the rolled-up model, we assume that a carbon nanocone comprises from one to five equilateral triangular panels, as shown in Figure 1. In addition, we assume that each triangular panel comprises infinite rows of unit equilateral triangles, and we denote each horizontal row of a triangular panel by the parameter *t*, which is chosen so that each horizontal line corresponds to some t∈N. We also define ϕ to be the angle between the mid-line of the triangle and any lattice point in the triangular panel, and therefore, −π/6⩽ϕ⩽π/6. For the purpose of these calculations, we may non-dimensionalise all lengths by the distance between adjacent points in the lattice σ which corresponds to the covalent bond length. To apply the rolled-up model described here to a physical structure, such as a carbon nanocone, we adopt a linear scaling using the carbon–carbon bond length σ≈1.4 Å, so that all dimensions need to be multiplied by this value. Likewise, for nanocones comprising other hexagonal materials, such as boron nitride nanocones, an appropriate value of σ may be used when applying this model. Thus, to determine the atomic locations of every atom, we must be able to locate the vertex of every point in the triangular panel relative to the conical vertex. This may be achieved by using the angle ϕ as defined above and also the distance (*s*) to any point from the vertex. From the definitions of *t* and ϕ, given previously, and using basic trigonometry we may derive

(1) $$s=(\sqrt{3}/2)t\sec\phi .$$

**Figure 2:**
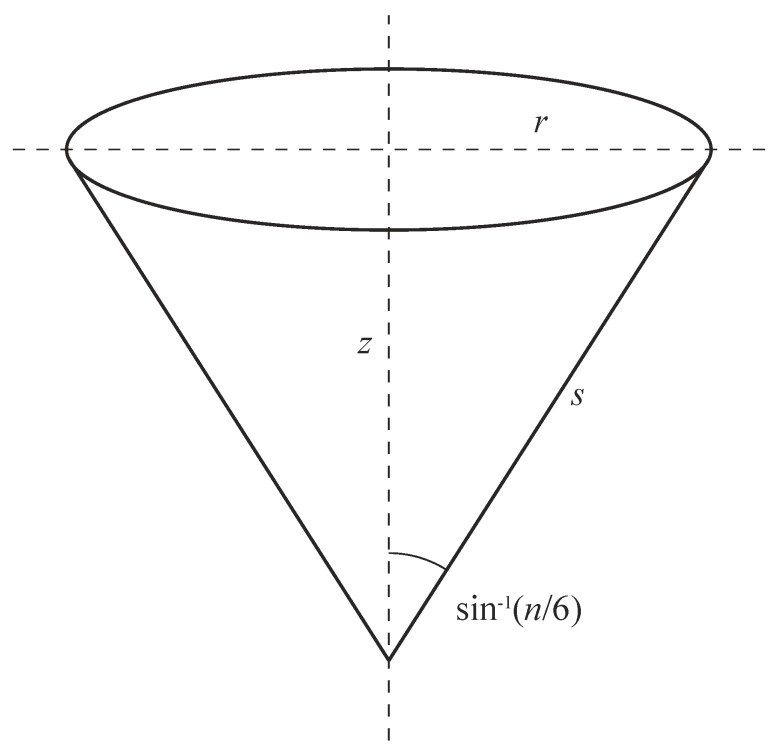
Geometry of the right circular cone showing the radius (*r*), height (*z*), and slant length from vertex *s* (the cone angle is determined from number of panels (*n*)).

The next step in constructing a cone is to map the points from the flat triangular panel(s) onto a right circular cone. An example of such a cone is given in Figure 2, where the slant length from vertex *s* is the same as the distance from the vertex to any point in the flat triangular panel defined above, and the parameters *r* and *z* denote the radius and height, respectively. Since the surface of the cone comprises an integer number of triangular panels (n∈{1,2,3,4,5}), then, using basic trigonometry, we can show that the conical angle (α/2) is given by $\alpha /2=\sin^{-1}\left(n/6\right)$. From this result, we may determine the rolled-up radius ($r_{0}$) and height ($z_{0}$) in terms of *s* and *n* by the formulae

(2) $$\begin{array}{cc} r_{0} & =ns/6, \end{array}$$

(3) $$\begin{array}{cc} z_{0} & =s{\left(1-n^{2}/36\right)}^{1/2}. \end{array}$$

Using these and (1), we are then able to give (2) and (3) in terms of *t* and ϕ as

(4) $$r_{0}=\frac{\sqrt{3}}{12}nt\sec\phi ,\;z_{0}=\frac{\sqrt{3}}{12}{\left(36-n^{2}\right)}^{1/2}t\sec\phi .$$

We now introduce the variable *u* which denotes the individual points on a single horizontal line. With reference to Figure 1, we see that every point on a single panel may then be determined from a unique pair of numbers ((t,u)) where *t* denotes the line and *u* denotes the point on that line such that u∈{0,1,2,…,t}. The angle ϕ is given by

(5) $$\phi =\tan^{-1}\left(\frac{2u-t}{\sqrt{3}t}\right).$$

The numbers (t,u) may also be used to determine the points in the panel which correspond to atomic locations and those which do not. To transform the triangular lattice into an hexagonal lattice (for the case of a carbon nanocone), one third of the points represent the holes at the centres of the hexagons and therefore, do not correspond to atoms, and the remaining two thirds of the lattice points then denote the locations of carbon atoms in the panel. With reference to Figure 1, we see that t+u=3k, where *k* is any integer so the lattice point denoted by (t,u) corresponds to a hole. Furthermore, from (5), it follows that

$$\sec\phi =\frac{2}{\sqrt{3}t}{\left(t^{2}-tu+u^{2}\right)}^{1/2},$$

and thus, from (4), we may derive

(6) $$\begin{array}{cc} r_{0} & =\frac{n}{6}{\left(t^{2}-tu+u^{2}\right)}^{1/2}, \end{array}$$

(7) $$\begin{array}{cc} z_{0} & =\frac{{\left(36-n^{2}\right)}^{1/2}}{6}{\left(t^{2}-tu+u^{2}\right)}^{1/2}. \end{array}$$

**Figure 3:**
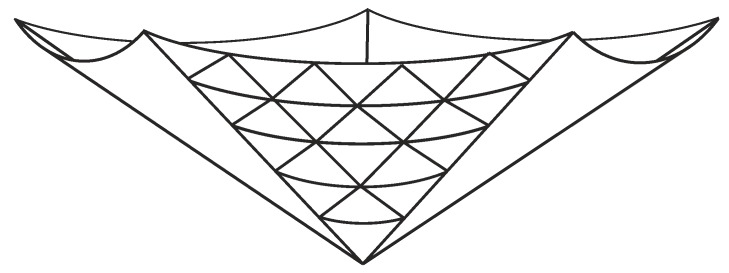
Example of a cone comprising five panels (triangular tessellation is shown on only one panel).

With all the points on a single panel being determined by the variables (t,u), and the radius ($r_{0}$) and height ($z_{0}$) being determined in terms of these variables, all that remains is to map these points to a system of three-dimensional Cartesian coordinates (x,y,z). With reference to Figure 3, it is clear that *n* copies of the panel are needed to complete the cone, and we align each panel such that its centre lies on the angle θ=2πk/n, where k∈{0,1,…,n−1}, and thus, the coordinates of a lattice point mapped onto the three-dimensional surface are given by

(8) $$x=r_{0}\cos\left(6\phi /n+\theta \right),\;y=r_{0}\sin\left(6\phi /n+\theta \right),\;z=z_{0},$$

where $r_{0}$ is given by (6), ϕ is given by (5), and the *z*-coordinate is precisely as given in (7).

**Figure 4:**
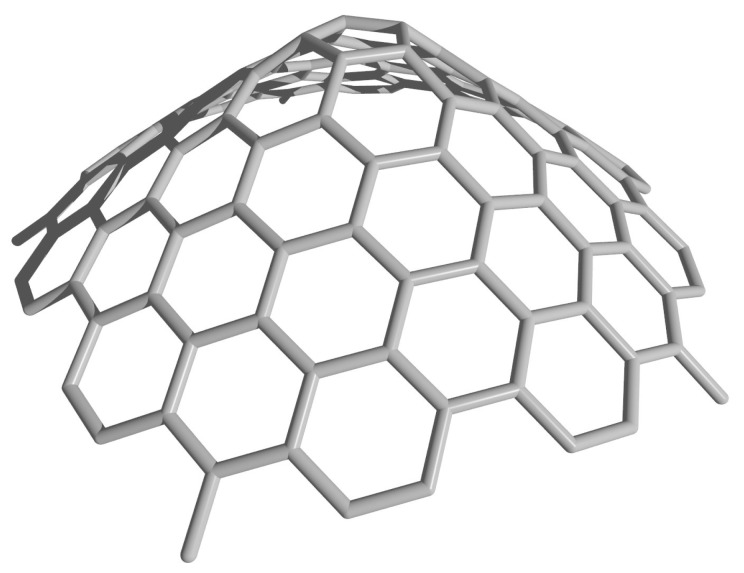
Cone constructed from the rolled-up model with n=5.

Therefore, by mapping points from triangular shaped panels onto a right circular cone, we are able to uniquely determine the coordinates of any point in the rolled-up model. An example of a cone constructed from the rolled-up model with n=5 is shown in Figure 4. However, in doing so, we compromise the assumption that all bond lengths are equal, since the bonds lying in planes including the on axis (for example, using the (t,u) notation, those between the points (t,0) and (t+1,0)) have no shortening due to curvature, yet all other bonds do have some curvature-induced shortening. The bonds most affected are those lying in planes perpendicular to the conical axis, and furthermore, those bonds lying nearer to the conical vertex are affected more than those further from the axis where the conical radius is larger. In the following section, we describe a new geometric model which makes some correction for this curvature-induced shortening.

## 4. Calculation

### New Geometric Model Formulation

The starting point for the new geometric model is to reconsider Equations (2) and (3) in an effort to prescribe relationships for *r* and *z* which take into account the curvature issues mentioned in the previous section. We remark that in the construction of this new model, we continue to use the parameter *s*, even though it no longer denotes the slant length from the vertex in the new model. Strictly speaking, the new model admits some freedom and does not describe a geometrically precise cone. Therefore, whenever *s* is used, it should be thought of as a parameter which only corresponds to the slant length in the case of the cone in the corresponding rolled-up model. As we shall show, the scale of *s* is preserved in the new model so that it represents distance along the profile of the cone.

As previously mentioned, the bonds lying in the planes perpendicular to the cone axis suffer the greatest curvature distortion in terms of the shortening of the Euclidean distance between lattice points. Therefore, a first step in formulating a corrected curvature model is to adjust the radius (*r*) so that these bonds are identical to the bond length. With reference to Figure 1, we see that the bonds in question are those that are bisected by the mid-line of the triangle and occur for every odd value for *t*. We denote the angle ϕ for these bonds as $\phi_{r}$, and from geometric considerations we can show that

(9) $$\phi_{r}=\sin^{-1}\left(1/2s\right).$$

Now, we consider a triangle lying in the plane perpendicular to the cone axis and containing one of these bonds, and we construct an isosceles triangle comprising the bond in question as its base and the point where the cone axis passes through the plane as the third vertex. From this, it is clear that the two equal sides of this triangle are of length *r* and the third side is unity. Furthermore, the angle between the equal sides is $2\phi_{r}$ which has been scaled by a factor of 6/n. Therefore, from the cosine rule we may write

$$1=2r^{2}\left[1-\cos\left(\frac{12\phi_{r}}{n}\right)\right],$$

which can be rearranged to give

$$r=\frac{1}{2}\mathrm{cosec}\left(\frac{6}{n}\phi_{r}\right),$$

and substituting (9) finally yields

(10) $$r=\frac{1}{2}\mathrm{cosec}\left(\frac{6}{n}\sin^{-1}\left(\frac{1}{2s}\right)\right).$$

Thus, we have derived an equation which approximates (2) for large values of *s*. This large *s* behaviour can be quickly established by considering the limit of *s* becoming large so that in that limit, $\sin^{-1}\left(1/2s\right)$ approaches 1/2s and likewise, $\mathrm{cosec}\left(3/sn\right)$ approaches sn/3. We also comment that while the derivation of (10) proceeds from discrete considerations, it can be applied for arbitrary real values of *s* and *n*, provided that care is taken to avoid the nonanalytic points. In particular, we note that the vertex itself corresponds to a value of s=0 in the rolled-up model, but in the new model, the radius is not defined for s=0. Further, we comment that the expression in (10) can be expanded as a series in terms of 1/s, and doing so yields

$$r=\frac{sn}{6}\left(1+\frac{36-n^{2}}{24n^{2}s^{2}}+\frac{\left(36-n^{2}\right)\left(17n^{2}+252\right)}{5760n^{4}s^{4}}\right)+O\left(\frac{1}{s^{5}}\right),$$

which shows that the leading order term is precisely the expression for $r_{0}$, and subsequent terms can be thought of as correction terms to the rolled-up conical radius.

While (10) is a compact expression, it does involve trigonometric functions (that is, transcendental functions). However, it is worth noting that for n∈{1,2,3,4}, (10) can be expressed explicitly as an algebraic function of *s*. For some particular values of *n* (most notably n=2), these relations are strikingly simple and are given by

$$\begin{array}{cc} n=1, & \;r=\frac{s^{6}}{{\left(4s^{2}-1\right)}^{1/2}\left(3s^{4}-4s^{2}+1\right)},\\ n=2, & \;r=\frac{s^{3}}{3s^{2}-1},\\ n=3, & \;r=\frac{s^{2}}{{\left(4s^{2}-1\right)}^{1/2}},\\ n=4, & \;r=\frac{s^{3/2}}{{\left[2s^{3}-\left(s^{2}-1\right){\left(4s^{2}-1\right)}^{1/2}\right]}^{1/2}}, \end{array}$$

and the derivations of these relations are given in the Appendix A. We note that when n=2, the relationship for *r* reduces to a very simple rational function of *s*.

The next step in the development of the new model is to derive a new relationship for *z*. If we consider the expressions from the rolled-up model given in (2) and (3), we notice that they satisfy the identity

(11) $${\left(\frac{dr_{0}}{ds}\right)}^{2}+{\left(\frac{dz_{0}}{ds}\right)}^{2}=1,$$

and this relationship is independent of the value of *n*. This result has an obvious geometric interpretation, which says the infinitesimals $dr_{0}$ and $dz_{0}$ are the perpendicular sides of a right-angle triangle with a hypotenuse (ds), which is to say that ds is the infinitesimal distance along the slanted profile of the cone. In this new model, we determine *z* by imposing that *s* continues to represent the slant length in the new model and therefore the relationship (11) continues to hold for *r* and *z*. In other words, we define *z* by the indefinite integral

(12) $$z=\int_{0}^{s}{\left\{1-{\left[\frac{d}{d\xi }\left(\frac{1}{2\sin\left(\frac{6}{n}\sin^{-1}\frac{1}{2\xi }\right)}\right)\right]}^{2}\right\}}^{1/2}\;d\xi ,$$

which satisfies the constraint that for s=0, z=0. Evaluating the derivative in (12) and simplifying yields

(13) $$z=\int_{0}^{s}{\left(1-\frac{9\cos^{2}\left(\frac{6}{n}\sin^{-1}\frac{1}{2\xi }\right)}{n^{2}\xi^{2}\left(4\xi^{2}-1\right)\sin^{4}\left(\frac{6}{n}\sin^{-1}\frac{1}{2\xi }\right)}\right)}^{1/2}\;d\xi .$$

However, the integrals (12) or (13) are not trivial to perform analytically and so, for the purposes of calculation, it is useful to calculate a series for *z* in terms of 1/s. By taking the asymptotic series for the integrand in (13) and integrating term by term, we find that

(14) $$z=\frac{s\sqrt{36-n^{2}}}{6}\left(1-\frac{1}{24s^{2}}-\frac{17n^{2}+192}{5760n^{2}s^{4}}\right)+O\left(\frac{1}{s^{5}}\right).$$

We comment that the leading order term is precisely the expression for $z_{0}$, and subsequent terms can be considered to be curvature-related corrections to the *z* coordinate. By this, we mean that the higher order terms in (14) arise directly from the attempt to accommodate changes in curvature close to the vertex. We also comment that the series or integrand converges absolutely for s>1 and thus, for all physically interesting values of *s*, the changing the order of the sum and integral is valid.

## 5. Results and Discussion

**Figure 5:**
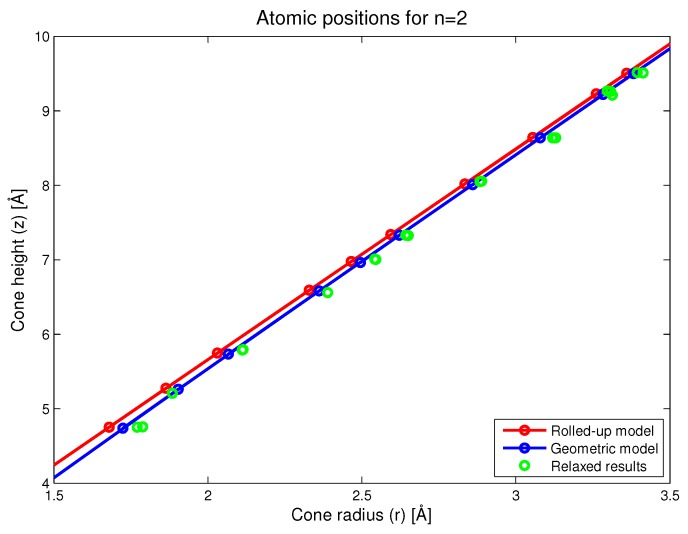
Atomic positions as predicted by the rolled-up, new geometric and simulation models.

In this section, we present the results for a nanocone with n=2. This species of nanocone was selected because it is a highly curved nanocone where curvature effects should be more readily apparent. Additionally, this structure also contains a notional rotational symmetry in the sense that every atom in the first panel can be paired with a corresponding atom in the second panel. The distance between two paired atoms provides a way of easily measuring the local radius of the cone for all such atom pairs. In Figure 5, we show the locations for atoms close to the conical vertex on the (r,z)-plane. Here, we see that the main difference in the predictions between the rolled-up and new geometric models is that *r* tends to be slightly larger in the new geometric model, meaning that the cones would tend to have a slightly puckered shape. The results of the simulation confirm that the new geometric model is closer to the resulting structure after relaxation than the conventional rolled-up model. In fact, it would appear that the new geometric model slightly underestimates the degree of puckering that results from the numerical relaxation process.

**Figure 6:**
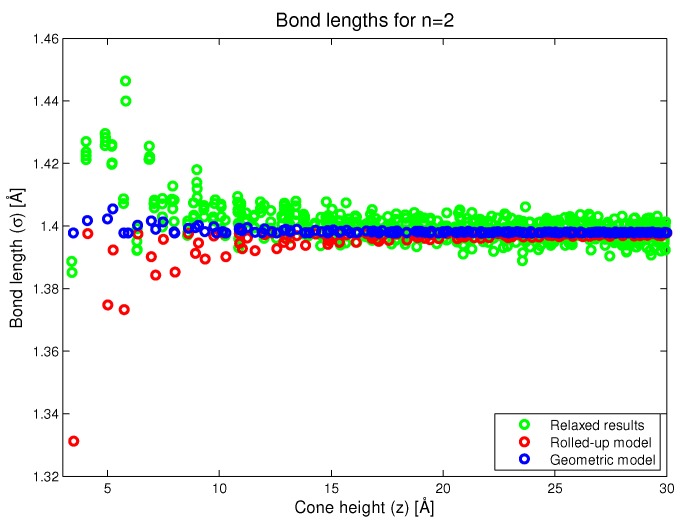
Bond lengths as predicted by the rolled-up, new geometric and simulation methods.

In Figure 6, we plot the carbon–carbon bond length as a function of conical height (*z*). In this figure, we see that the rolled-up model predicts that as we approach the vertex, certain bond lengths will be substantially less than the bond length in the flat graphene. This is not an intentional prediction of the model but rather, an artifact of the rolled-up process. In the case of the new geometric model, we try as much as possible to make all the bond lengths the same, but this is not completely satisfied, and we observe that some bonds closer to the vertex are larger than the bond length in the flat graphene. However, it should be noted that this is an artifact of the assumptions of the geometric model and not an intentional prediction of the model. However, the magnitude of the lengthening is generally less than the degree of shortening in the rolled-up case. The results of the simulation show a definite trend of bond lengthening closer to the conical vertex which is probably due to repulsive interactions between atoms on opposite sides of the cone. We comment that the primary effect of approaching the cone vertex is an increased localisation of the double bonding and a reduction in the aromaticity, approaching fullerene behaviour. This means that bonds become increasingly single or double in character. This is revealed in the relaxed numerical results in Figure 6, and it is an effect that is not taken into account in the new geometric model proposed here.

**Figure 7:**
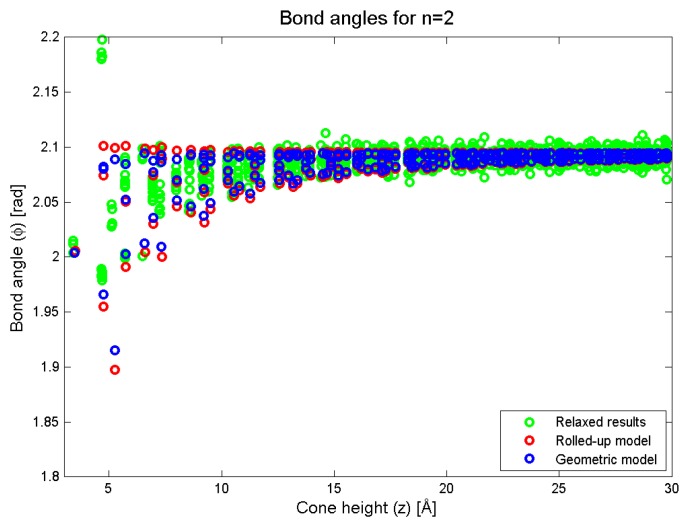
Bond angles as predicted by the rolled-up, new geometric and simulation methods.

In Figure 7, we plot the bond angles as a function of the conical height (*z*). In this figure, we see that the two models are in approximate agreement, although the variance in the bond angle for a particular value of *z* is lower than that in the geometric prediction. The results of the simulation show that the trend of a decreasing bond angle is also matched in the simulation. For small values of *z* (less than 10 Å), the variance in the simulation data is even lower than that predicted by the new geometric model which means that once again, the geometric model is capturing the right trend but slightly underestimating the numerical picture.

## 6. Conclusions

In this paper, we proposed a new geometric model for carbon nanocones, and we examined the fine geometric structure using molecular simulation as a way of assessing two models for the nanocone geometric structure: the classical rolled-up model and a new geometric model proposed here by the authors. The results indicate that the key features of the geometric structure, such as the conical radius (*r*) as a function of the distance from the vertex (*z*) and the variation in bond angle, exhibit trends which are predicted by the geometric model. However, the new geometric model slightly underestimates both of these effects. The simulation also shows a marked increase in bond length which is also an artifact of the geometric model. Again, the magnitude of this effect is underestimated by the geometric model. A future direction of research might be to modify the geometric model to accurately capture the bond lengthening effect which might yield even better predictions for atomic locations and bond angle variability. An additional future direction may be the introduction of a Burgers vector which may enable the model to be applied to nanocones with a screw dislocation stacking fault.

We further comment that the model proposed here has certain limitations in its applicability. Firstly, the curvature effect addressed is a secondary one, since, as the distance from the cone tip increases, the effect rapidly decays. Hence, it is only a relatively small correction as the tip is approached, and as the above numerical results indicate, it can be accommodated by numerical modelling with relatively few molecular dynamics iterations, or via the first few steps in geometric optimisation using a quantum chemical code. Nevertheless, the new model proposed here has the potential to save computational time in such alternatives. Secondly, the new model only applies to a very limited range of cone geometries, namely, symmetrical cones and in particular, those with a symmetrical vertex. We avoided this difficulty by simply removing the cone vertex from the analysis to avoid addressing this question. For example, the new model does not apply when a pentagon associated with the nanocone vertex does not lie exactly on the cone axis. Of course, this significantly reduces the utility of the model, and for example, in the case of four panel cones with a square at the cone vertex, the model does not address the necessary structural rearrangements in order to convert such a vertex into one containing only pentagons. Such structural rearrangements have significant effects on the bond lengths out to at least 1 nm, and this is the range over which the current model shows the most deviation from the conventional model. However, our results do apply for all five values of *n*, in particular, for n=2, as shown in Figure 6 and Figure 7 where the cone vertex has been replaced by hydrogen atoms, and we emphasise that the model presented here focuses on the structure leading up to the vertex, rather than the vertex structure itself.

## Appendix A. Algebraic Formulae for r

In this appendix, we outline the derivation of algebraic expressions for *r* for n∈{1,2,3,4}. The method for removing the trigonometric functions from (10) is to use the part angle and multiple angle formulae for the sine function to produce an expression with terms of the forms $\sin\left(\sin^{-1}x\right)$ and $\cos\left(\sin^{-1}x\right)$, and then, these expressions are replaced with *x* and $\sqrt{1-x^{2}}$, respectively. We comment that in this processm we are dealing with essentially multivalued functions. However, for our purposes, we require only the value which is usually the positive root and which includes zero in the range of possible values.

For n=1 m we make use of the trigonometric identity

(A1) $$\begin{array}{c} \sin6\theta =6\cos^{5}\theta \sin\theta -20\cos^{3}\theta \sin^{3}\theta +6\cos\theta \sin^{5}\theta , \end{array}$$

so that

$$\begin{array}{cc} r & ={\left[2\sin\left(6\sin^{-1}\frac{1}{2s}\right)\right]}^{-1}\\ & ={\left[2\left(6\frac{{\left(4s^{2}-1\right)}^{5/2}}{{\left(2s\right)}^{6}}-20\frac{{\left(4s^{2}-1\right)}^{3/2}}{{\left(2s\right)}^{6}}+6\frac{{\left(4s^{2}-1\right)}^{1/2}}{{\left(2s\right)}^{6}}\right)\right]}^{-1}\\ & =\frac{16s^{6}}{{\left(4s^{2}-1\right)}^{1/2}\left[3{\left(4s^{2}-1\right)}^{2}-10\left(4s^{2}-1\right)+3\right]}\\ & =\frac{s^{6}}{{\left(4s^{2}-1\right)}^{1/2}\left(3s^{4}-4s^{2}+1\right)}. \end{array}$$

On utilising the trigonometric identity

(A2) $$\begin{array}{c} \sin3\theta =3\cos^{2}\theta \sin\theta -\sin^{3}\theta , \end{array}$$

and substituting into (10), for n=2, we obtain

$$\begin{array}{cc} r & ={\left[2\sin\left(3\sin^{-1}\frac{1}{2s}\right)\right]}^{-1}\\ & ={\left[2\left(3\frac{4s^{2}-1}{{\left(2s\right)}^{3}}-\frac{1}{{\left(2s\right)}^{3}}\right)\right]}^{-1}\\ & =\frac{4s^{3}}{12s^{2}-3-1}\\ & =\frac{s^{3}}{3s^{2}-1}. \end{array}$$

The only other case which can be evaluated analytically using just the multiple angle formulae is n=3. Here, we employ the simple trigonometric identity

$$\begin{array}{c} \sin2\theta =2\cos\theta \sin\theta , \end{array}$$

which gives

$$\begin{array}{c} r={\left[2\sin\left(2\sin^{-1}\frac{1}{2s}\right)\right]}^{-1}={\left[4\frac{{\left(4s^{2}-1\right)}^{2/3}}{{\left(2s\right)}^{2}}\right]}^{-1}=\frac{s^{2}}{{\left(4s^{2}-1\right)}^{1/2}}. \end{array}$$

In the n=4 case, we must use a combination of the trigonometric identity (A2) and the half-angle formula

$$\begin{array}{c} \sin\frac{\theta }{2}={\left(\frac{1-\cos\theta }{2}\right)}^{1/2}, \end{array}$$

and we are interested in the positive square root. Thus, by substituting into (10), we may derive

$$\begin{array}{cc} r & ={\left[2\sin\left(\frac{3}{2}\sin^{-1}\frac{1}{2s}\right)\right]}^{-1}={\left\{2{\left[\frac{1}{2}-\frac{{\left(4s^{2}-1\right)}^{3/2}}{2{\left(2s\right)}^{3}}+3\frac{{\left(4s^{2}-1\right)}^{1/2}}{2{\left(2s\right)}^{3}}\right]}^{1/2}\right\}}^{-1}\\ & =\frac{2s^{2}}{{\left[8s^{4}-s{\left(4s^{2}-1\right)}^{3/2}+3s{\left(4s^{2}-1\right)}^{1/2}\right]}^{1/2}}=\frac{s^{2}}{{\left[2s^{4}+s\left(1-s^{2}\right){\left(4s^{2}-1\right)}^{1/2}\right]}^{1/2}}. \end{array}$$

Finally, for n=5, we comment that an algebraic expression with real coefficients is possible but cumbersome, and we note that a general *n* algebraic expression for *r* is given by

$$\begin{array}{cc} r & =\frac{i{\left(2s\right)}^{6/n}}{{\left(\sqrt{4s^{2}-1}+i\right)}^{6/n}-{\left(\sqrt{4s^{2}-1}-i\right)}^{6/n}}, \end{array}$$

which holds for all n∈{1,2,3,4,5}, and from which the simple expressions for n=1, 2 and 3 can be seen to originate immediately.
